# 3D Printed Tacrolimus Rectal Formulations Ameliorate Colitis in an Experimental Animal Model of Inflammatory Bowel Disease

**DOI:** 10.3390/biomedicines8120563

**Published:** 2020-12-02

**Authors:** Iria Seoane-Viaño, Noemí Gómez-Lado, Héctor Lázare-Iglesias, Xurxo García-Otero, José Ramón Antúnez-López, Álvaro Ruibal, Juan Jesús Varela-Correa, Pablo Aguiar, Abdul W. Basit, Francisco J. Otero-Espinar, Miguel González-Barcia, Alvaro Goyanes, Asteria Luzardo-Álvarez, Anxo Fernández-Ferreiro

**Affiliations:** 1 Department of Pharmacology, Pharmacy and Pharmaceutical Technology, Faculty of Pharmacy, University of Santiago de Compostela (USC), 15782 Santiago de Compostela, Spain; i.seoane.viano@usc.es (I.S.-V.); xurxo.garcia.otero@gmail.com (X.G.-O.); francisco.otero@usc.es (F.J.O.-E.); 2 Paraquasil Group, Health Research Institute of Santiago de Compostela (IDIS), 15706 Santiago de Compostela, Spain; 3 Nuclear Medicine Department and Molecular Imaging Group, University Clinical Hospital (CHUS) and Health Research Institute of Santiago de Compostela (IDIS), 15706 Santiago de Compostela, Spain; noegola@yahoo.es (N.G.-L.); alvaro.ruibal.morell@sergas.es (Á.R.); pablo.aguiar.fernandez@sergas.es (P.A.); 4 Pathology Department, University Clinical Hospital Santiago de Compostela (SERGAS) (CHUS), 15706 Santiago de Compostela, Spain; hectorlazare@gmail.com (H.L.-I.); Jose.Ramon.Antunez.Lopez@sergas.es (J.R.A.-L.); 5 Tejerina Foundation, José Abascal 40, 28003 Madrid, Spain; 6 Pharmacy Department, University Hospital Ourense (SERGAS), Calle Ramón Puga Noguerol 54, 32005 Ourense, Spain; Juan.Jesus.Varela.Correa@sergas.es; 7 FabRx Ltd., 3 Romney Road, Ashford, Kent TN24 0RW, UK; a.basit@ucl.ac.uk; 8 Department of Pharmaceutics, UCL School of Pharmacy, University College London, 29-39 Brunswick Square, London WC1N 1AX, UK; 9 Pharmacy Department, University Clinical Hospital Santiago de Compostela (SERGAS) (CHUS), 15706 Santiago de Compostela, Spain; miguel.gonzalez.barcia@sergas.es; 10 Departamento de Farmacología, Farmacia y Tecnología Farmacéutica, I+D Farma Group (GI-1645), Universidade de Santiago de Compostela, 15782 Santiago de Compostela, Spain; 11 Clinical Pharmacology Group, Health Research Institute of Santiago de Compostela (IDIS), 15706 Santiago de Compostela, Spain

**Keywords:** three-dimensional printing, PET/CT imaging, rectal drug delivery, ulcerative colitis, TNBS rat model, M3dimaker, 3D printed drug products, personalized medicines and pharmaceuticals

## Abstract

The aim of this study was to fabricate novel self-supporting tacrolimus suppositories using semisolid extrusion 3-dimensional printing (3DP) and to investigate their efficacy in an experimental model of inflammatory bowel disease. Blends of Gelucire 44/14 and coconut oil were employed as lipid excipients to obtain suppository formulations with self-emulsifying properties, which were then tested in a TNBS (2,4,6-trinitrobenzenesulfonic acid) induced rat colitis model. Disease activity was monitored using PET/CT medical imaging; maximum standardized uptake values (SUV_max_), a measure of tissue radiotracer accumulation rate, together with body weight changes and histological assessments, were used as inflammatory indices to monitor treatment efficacy. Following tacrolimus treatment, a significant reduction in SUV_max_ was observed on days 7 and 10 in the rat colon sections compared to non-treated animals. Histological analysis using Nancy index confirmed disease remission. Moreover, statistical analysis showed a positive correlation (R^2^ = 71.48%) between SUV_max_ values and weight changes over time. Overall, this study demonstrates the effectiveness of 3D printed tacrolimus suppositories to ameliorate colitis and highlights the utility of non-invasive PET/CT imaging to evaluate new therapies in the preclinical area.

## 1. Introduction

Inflammatory bowel disease (IBD) is a chronic disorder of unknown aetiology that causes prolonged inflammation of the gastrointestinal (GI) tract. The two main types of IBD are Crohn’s disease (CD) and ulcerative colitis (UC), being highly heterogenic with regard to activity, site and behavior of the disease [1]. Although the aetiology of IBD remains to be fully elucidated, an interplay of luminal microbiota, external environment and disturbances in the immune responses are hypothesized to trigger the onset of the disease in a genetically susceptible host [2]. Conventional treatment of IBD is based on the topical and systemic use of 5-aminosalicylic acid, azathioprine and steroids. However, this first line of treatment does not always achieve remission of the disease. When this happens, biological treatments such as infliximab, tocilizumab or ustekinumab are used [3,4]. In cases where even these commercialized drugs are not sufficient to treat symptoms, pharmaceutical compounding is the last alternative to treat these patients [5].

Tacrolimus, a macrolide antibiotic with potent immunosuppressive properties [6], represent another treatment option for medication-resistant IBD, which can be administered orally, intravenously or rectally [7]. This drug induces a rapid clinical response and mucosal healing in hospitalized patients with steroid-refractory UC and its use is also supported in CD refractory to conventional therapies [8]. However, administration of oral or intravenous tacrolimus is related to toxicity and systemic adverse effects such as hypertension and renal impairment, limiting its use. These adverse effects are highly related to the trough serum levels of tacrolimus, which should be monitored [9]. With the aim of reducing systemic side effects, topical therapy is often employed [10], mainly in the form of suppositories, but enemas and ointments are also used. Each type of formulation can reach different areas of the colon. Depending on the volume administered, enemas can reach the splenic flexure, while suppositories are more intended for local drug release into the rectum [11].

This rectal route of administration presents several advantages, such as the possibility to locally treat some conditions and the minimization of the first-pass effect if the drug is administered to the lower part of the rectum. In the case of tacrolimus therapy, although tacrolimus is absorbed through the rectal mucosa, the systemic levels of the drug are low and the number of adverse events is limited [12]. However, tacrolimus suppositories are not commercially available, so they are often compounded in hospital pharmacy settings [6].

Preclinical animal models of colitis, such as the TNBS (2,4,6-trinitrobenzenesulfonic acid) [13] and dextran sodium sulfate (DSS) [14] induced colitis models could be used to demonstrate efficacy of treatments and the ability of pharmaceutical products to reach the area of interest for their therapeutic activity [15]. The beneficial effect of tacrolimus in an animal model of DSS colitis was first proposed in 1995 [16]. Since then, other studies have reported the therapeutic potential of tacrolimus in the treatment of IBD. For instance, a study using tacrolimus entrapped into nanoparticles found that a more beneficial effect is obtained when the drug was subcutaneously, rather than orally, administered [17]. Another study in a mouse DSS model showed that intrarectal administration of tacrolimus exerts a therapeutic effect through induction of apoptosis in activated macrophages [18]. However, there are still no studies in animal models of colitis that use tacrolimus suppositories to treat experimental IBD. It is necessary to emphasize that although IBD animal models allow us to better understand the complex mechanisms involved in chronic intestinal inflammation and its remission, they only partially reflect the complexity of the human disease [19]. Medical imaging techniques commonly used in clinical practice [20] represent a useful tool for noninvasively evaluating disease progression before and after treatment administration in preclinical animal studies [15,21]. In particular, PET/CT (positron emission tomography/computed tomography) technology, allows repeated measurements using the same animal, thus reducing the number of animals needed [22], and has proven useful for the assessment of experimental colitis in rats [13,20]. Moreover, the maximum standardized uptake value (SUV_max_), a common biomarker used in current clinical practice for the assessment of inflammatory processes, can be also used for evaluating disease progression and treatment response in animal models of IBD [23,24]. Thus, the PET/CT imaging technique could be used to monitor disease activity before and after treatment with tacrolimus suppositories.

These suppositories are to be administered to small animals as rodents, so it is necessary to prepare them in an appropriate size and shape. To avoid the need for a mold specifically designed to meet these requirements, three-dimensional printing (3DP) technology can offer an alternative for the manufacture of small batches of suppositories, as demonstrated in a previous study were SSE was used to print human suppositories [25]. The implementation of 3DP in clinical practice allows one to personalize the dose to the patient’s requirements, which ultimately leads to better outcomes for patients. In the preclinical field, this additive manufacturing technology enables the production of small devices with a size and dose adapted to the needs of the preclinical study [26]. In particular, semisolid extrusion (SSE) 3DP technology is based on the deposition of semisolid materials (gel or pastes) in sequential layers through a syringe-based tool-head nozzle to create the 3D object, and it is highly relevant to print objects using soft materials [27,28,29]. SSE was the first 3DP technology used for the preparation of personalized dose printlets (3D printed tablets) in a hospital setting for the treatment of a rare metabolic disease [30]. Moreover, SSE was also used for printing lipid-based formulations, as solid self-microemulsifying drug delivery systems (S-SMEDDS) intended for oral administration [31]. Self-emulsifying drug delivery systems (SEDDS) and self-microemulsifying drug delivery systems (S-SMEDDS) are lipid-based isotropic mixtures of oils, surfactants and cosurfactants that form kinetically stable oil-in-water (O/W) emulsions under mild agitation [32]. This approach is especially useful for enhancing drug solubility of poorly water-soluble drugs, which are solubilized in the small drops of oil [33]. The low oral bioavailability of tacrolimus and its poor water solubility made it a suitable candidate for inclusion in a 3D printed lipid-based suppository [34].

The aim of this study was to evaluate the therapeutic activity of 3D printed self-emulsifying suppositories (SES) loaded with tacrolimus for the treatment of experimental IBD in a previously developed and validated TNBS colitis animal model [13]. Moreover, the in vivo disintegration time and distribution of the formulations was studied using barium sulphate as a contrast agent for computed tomography (CT) imaging. Apart from using PET/CT imaging for monitoring disease progression and therapeutic response in the animal model, histological analyses were carried out to confirm the results obtained from the quantification of SUV_max_ values from PET images.

## 2. Experimental Section

### 2.1. Materials

Tacrolimus was purchased from Guinama S.L.U., Valencia, Spain. Coconut oil was obtained from Acofarma, Madrid, Spain. Barium sulphate Reagent Grade, 99%, was obtained from Honeywell, London, UK. Gelucire 44/14 was kindly donated by Gattefosse España SA, Madrid, Spain. TNBS (2,4,6-trinitrobenzenesulfonic acid) was purchased from Sigma-Aldrich Company Ltd., Madrid, Spain. Ethanol absolute was obtained from VWR International S.A.S., Briare, France. NaCl 0.9% B. Braun was purchased from Braun Medical Inc. Barcelona, Spain. Ultravist^®^ 300 mg/mL was purchased from Bayer Hispania S.L., Barcelona, Spain.

### 2.2. Methods

#### 2.2.1. 3D Design

**Figure 1:**
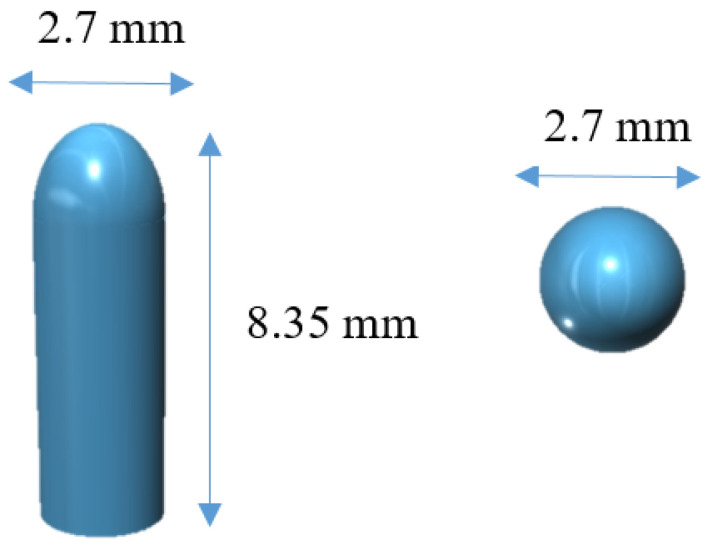
3D model of the suppositories from different angles.

The software 123D Design (Autodesk Inc., Manchester, NH, USA) was used to design the templates of the suppositories, size 2.7 mm diameter × 8.35 mm height (Figure 1).

#### 2.2.2. Semisolid Extrusion 3D Printing

The mixture of lipid excipients and drug was selected from a previous study [25] where different combinations of lipid excipients were tested. The mixture composed of 79.55% of Gelucire 44/14, 19.55% of coconut oil and 0.9% of tacrolimus was mixed in a glass beaker and placed on a heating plate. The tacrolimus dose of 2 mg/kg body weight was selected according to published literature [35]. The mixture was heated up to the melting point of the mixture (42 °C) under magnetic stirring until the complete solubilization of the drug in the lipid excipients. The mass was immediately transferred to a 5 mL extrusion syringe with a tapered extrusion tip (0.9 mm orifice) and allowed to solidify at room temperature. Then, the syringe was placed into the pharmaceutical 3D printer with the semisolid extrusion tool (M3DIMAKER, FabRx Ltd., London, UK). The 3D model of the suppositories was designed using the 123D Design (Autodesk Inc., USA), which was subsequently exported as a .stl file into a 3D printer software (Repetier host v. 2.1.3, Hot-World GmbH & Co. KG, Willich, Germany). The selected 3D geometry was a bullet shaped printlet (2.7 mm diameter × 8.35 height). The printer settings in the Repetier Host software were as follows: 25 mm/s flow speed; extrusion temperature 42 °C; 0.2 mm layer height; 2.4 mm shell thickness; 0.9 mm nozzle size, and printed in the horizontal position. Finally, 3D printed suppositories were allowed to solidify at room temperature and subsequently stored in refrigeration at 4 °C.

#### 2.2.3. Characterization of the 3D Printed Suppositories

##### Drug Loading

The content uniformity of tacrolimus in the 3DP suppositories was determined in triplicate using high performance liquid chromatography (HPLC). Each suppository of approximately 50 mg was dissolved into 50 mL of ethanol absolute at room temperature with magnetic stirring to form a clear transparent solution. The amount of drug in solution was determined using an Agilent 1260 series HPLC system (Agilent Technologies, Wilmington, DE, USA) equipped with diode array detector HS, a solvent delivery quaternary pump system, maximum pressure of 400 bar and an autosampler with thermostat. The software model OpenLAB CDS 3D UV (PDA) was used for the data processing. The analysis was performed in an isocratic method. The column used was a Poroshell 120, EC-C18 (4.6 mm × 100 mm, 4 μm) and at a temperature of 60 °C. The mobile phase was water-acetonitrile (35:65 v/v) using a flow rate of 1.5 mL·min^−1^. A wavelength of 210 nm was employed for the quantification of tacrolimus. The volume of the injected sample was 10 μL and the retention time was 3.3 min. Each sample was assayed in triplicate.

##### In Vitro Disintegration Time of 3D Printed Suppositories

The test was performed in distilled water at 37 °C using the U.S.P. disintegration apparatus slightly modified to meet the requirements of the method described in the European Pharmacopeia [36]. Each suppository was placed between the two perforated plates of the basket, which was inserted into a transparent plastic sleeve. The suppository disintegration rig was then placed in the glass beaker containing 1 L of distilled water at 37 °C. The mean values were calculated from three parallel measurements for the 3D printed suppositories with barium sulphate and three measurements for the suppositories without barium sulphate. The Mann–Whitney non-parametric test was used to evaluate the statistical significance of the difference between the obtained results from the two formulations. (GraphPad Prism version 7.0 (GraphPad Software, San Diego, CA, USA), *p* < 0.05 was considered statistically significant).

##### In Vitro Drug Release

In order to obtain the in vitro drug release profiles, the 3DP suppositories were placed into glass vials with 10 mL phosphate buffer (0.1 M) pH 8 under controlled conditions of agitation (100 rpm) and temperature (37 °C) using an orbital shaker (Heidolph Unimax 1010) to simulate conditions of the colon lumen. At appropriate time intervals, 1 mL aliquots were withdrawn and 1 mL of fresh medium was added to the vials. To solubilize the tacrolimus that could be trapped inside the lipid droplets, 0.5 mL of ethanol absolute were added to the aliquots. Then, the samples were centrifuged for 30 min at 12,500 rpm and the supernatant was collected. The samples were analyzed by HPLC as described earlier.

Tacrolimus release profile from suppositories could be fitted to the Gompertz growth model (Equation (1)):

(1) $$\%\;released=\%\max\;{\left(\frac{{\%}_{min}}{{\%}_{max}}\right)}^{e^{-kt}}$$

where %max is the maximum percentage of drug released, %min is the minimum percentage of drug released and *k* is a constant. The Gompertz growth model was fitted to the drug release profile by non-linear regression analysis using the program GraphPad Prism (version 7.0). The Gompertz growth model is a logistic function useful to describe S-shaped curves composed by an induction phase, a linear increase and a saturation or depletion end process.

##### In Vivo Disintegration Time of 3D Printed Suppositories

The in vivo disintegration time of the 3D printed suppositories was investigated by CT imaging in four Sprague-Dawley rats (average weight of 250 ± 25 g). Barium sulphate, used as a contrast agent, was dissolved in the mixture of lipid excipients and drug (30% w/w) and the printing process was carried out as described previously. The 3D printed suppositories were inserted intrarectally in the rats under anesthesia (2% isoflurane) and CT scans were performed immediately after administration and 20 and 50 min post-administration.

#### 2.2.4. Inflammatory Bowel Disease (IBD) Animal Model

These studies were carried out on male Sprague-Dawley rats (average weight of 250 ± 25 g) supplied by the animal facility at the University of Santiago de Compostela. During the experiments, animals were kept in individual cages under controlled temperature (22 ± 1 °C) and humidity (60% ± 5%) conditions, with day–night cycles regulated by artificial light (12/12 h) and fed ad libitum. All animal experiments complied the ARRIVE guidelines [37] and were carried out in accordance with the Spanish and European Union (UE) directive for animal experiments RD53/2013 and 2010/63/EU. Experiments were approved by the Galician Network Committee for Ethics Research (IDIS/2007-17, 12-07-2017). The TNBS-induced IBD animal model was obtained following the method previously described by Morris et al. [38]. All animals were fasted for 18 h before rectal administration of TNBS in a dose of 50 mg/kg body weight dissolved in ethanol 50% (v/v), through a catheter inserted rectally into the colon until reaching 8 cm proximal to the anus, under isoflurane anesthesia (2%). Then, they were kept in a vertical position for 1 min to prevent leakage of the intracolonic instillation. Finally, animals were returned to their cages with free access to food and water.

#### 2.2.5. Experimental Design

##### Assessment of the Efficacy of Tacrolimus Treatment

Tacrolimus suppositories were inserted daily into the rectum of 6 rats at a dose of 2 mg/kg under isoflurane anesthesia (2%). The treatment was started 3 days after induction of colitis and was administered until day 15, when the animals were sacrificed. [^18^F]FDG PET/CT scans were carried out before the IBD induction (basal condition) and 1, 3, 7, 10, 13 and 15 days post-TNBS administration. All the animals were daily weighted. Data from the control and non-treated group was already published in a previous study from this research group following the same protocol as for the present study [13]. The control group was composed by healthy animals that received a daily administration of intrarectal saline solution from day three after starting the experiment. Non-treated group was composed of animals with experimental colitis that did not receive any treatment.

##### PET/CT Acquisition and Evaluation

PET/CT images were acquired using an Albira PET/CT Preclinical Imaging System (Bruker Biospin, Woodbridge, CT, USA). The PET subsystem comprises three rings of eight compact modules based on monolithic crystals coupled to multianode photomultiplier tubes (MAPMTs), forming an octagon with an axial field of view (FOV) of 8 cm × 14.8 cm (transaxial and axial directions respectively). This subsystem generates PET images with spatial resolution of 1.2 mm and a sensitivity of 10%. The CT subsystem consists of a microfocus X-ray tube of 50 kVp, a CsI scintillator 2D pixelated flat panel detector and a FOV of 5.2 cm × 5.2 cm, generating images of 90 µm spatial resolution.

The animals were anesthetized with 2% isoflurane until they were unconscious and 12 ± 1 MBq of [^18^F]FDG were injected in the tail vein of each animal. After 40 min, 2 mL of Iopromide Ultravist^®^ 300 mg/mL (CT contrast agent) was administered intrarectally via a catheter inserted 8 cm proximal to the anus under isoflurane anesthesia. Then, PET/CT static acquisitions were performed, consisting of 20 min PET scan followed by a 20 min CT scan. PET images were reconstructed using the maximum likelihood expectation maximization (MLEM) algorithm with 12 iterations and an image pixel size of 0.5 × 0.5 × 0.5 mm^3^, including scatter and random coincidences and no attenuation correction. The CT acquisition parameters were 35 kV for a tube current of 200 A with 250 projections per bed and the reconstructed images had a pixel size of 0.25 x 0.25 x 0.25 mm^3^. The FOV of the PET scan focused from the upper part of the lungs to the lower extremities of the animal and the FOV of CT scan was focused on the abdominal region.

All images were analyzed using AMIDE software (amide.sourceforge.net; DHI Group, Inc., New York, NY, USA). Fused [^18^F]FDG PET/CT images were used to define three different regions of the rat colon (ascending, transverse and descending regions). Then, quantitative analysis was carried out by using circularly delineated regions of interest (ROIs) on CT images following longitudinal colon sections at ascending, transverse and descending regions. The cylindrical ROIs dimensions ranged from 5 to 15 mm in diameter (fitting to the diameter of the colon section) and 1 mm in length. Subsequently, the ROIs were transferred to PET images in order to calculate the maximum [^18^F]FDG uptake value. Finally, the standardized uptake value (SUV_max_) was calculated as the maximum [^18^F]FDG uptake value normalized by the injected activity and the body weight of the animal. The injected [^18^F]FDG activity was estimated by subtracting the extravasated activity in the tail.

Furthermore, the mean rat recovering time (MRRT) was calculated using an equation similar to that used to calculate MRT (mean residence time) in noncompartmental pharmacokinetic analysis [39]. Like MRT, MRRT represents the average time it takes for a rat to regain the baseline SUV_max_ values, and therefore helps interpret the effect of the drugs in the colon. MRRT was calculated using the following equation:

(2) $$MRRT=\frac{\int_{0}^{t}tSUV_{max}\left(t\right)dt}{\int_{0}^{t}SUV_{max}\left(t\right)dt}=\frac{AUMC}{AUC}$$

where AUC is the area under the curve SUV_max_ versus time and AUMC the area under the first moment curve SUV_max_ × time versus time.

Statistical analysis was carried out using a two-way analysis of the variance (ANOVA) including as factors time after induction of TNBS colitis and treatment. The data for non-treated animals were obtained from the previous work of this group [13] to compare the previous results with those obtained in the present study for animals treated with tacrolimus. Furthermore, Sidak’s multiple comparisons test was carried out for comparing SUV_max_ values over time. (GraphPad Prism (version 7.0, CA, USA), *p* < 0.05 was considered statistically significant).

##### Macroscopic Evaluation and Histopathology

The animals were sacrificed by CO_2_ inhalation (60–70%) in a euthanasia chamber on day 15 post-TNBS administration. Colons were removed, macroscopically examined and subsequently photographed. Afterwards, they were individually fixed in 10% formalin and dehydrated, paraffin embedded, sectioned in slices with 4 μm thickness and stained with H&E (hematoxylin and eosin). The samples were blindly evaluated by a digestive pathologist using a Zeiss^®^ microscope. The Nancy histological index was used as a reference score for the histological disease activity [40,41]. The activity was scored in five grades as follows: (Grade 4)—Ulceration of colonic mucosa with inflamed granulation tissue. (Grade 3)—Presence of multiple clusters of neutrophils in lamina propria and epithelium and the acute inflammatory cells infiltrate is moderate to severe. (Grade 2)—Presence of few neutrophils in lamina propria and in epithelium and mild acute inflammatory cells infiltrate. (Grade 1)—Chronic inflammatory infiltrate with no acute inflammatory infiltrate. (Grade 0)—No increase in chronic inflammatory cell number or absence of histological significant disease.

Statistical analysis was carried out using the Mann–Whitney nonparametric test to evaluate the differences between Nancy scores obtained in the ascending, transverse and descending regions of tacrolimus group and non-treated group. GraphPad Prism (version 7.0, CA, USA), *p* < 0.05 was considered statistically significant.

## 3. Results and Discussion

### 3.1. Characterization of 3D Printed Suppositories

In the study herein semisolid extrusion 3DP was used to prepare 3D printed suppositories loaded with the drug tacrolimus and subsequently test the feasibility of this approach to treat experimental colitis in a TNBS rat model.

**Figure 2:**
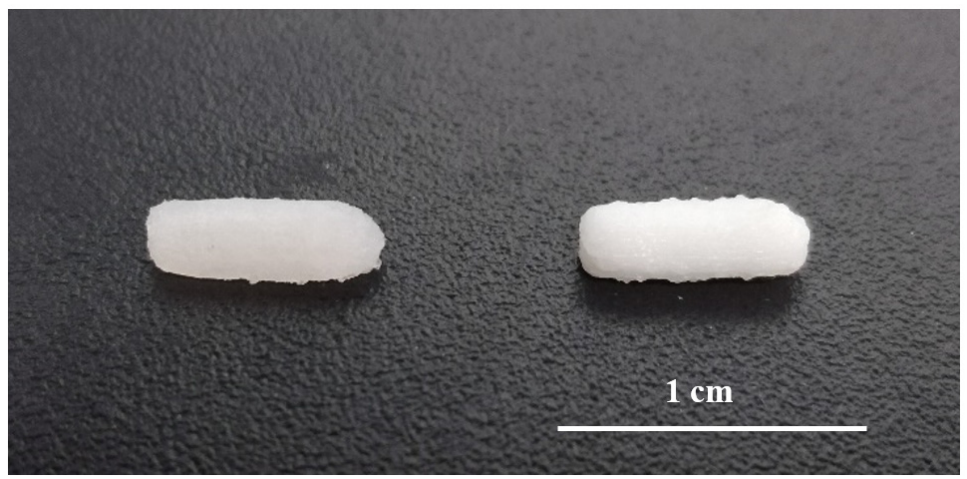
Picture of 3D printed suppositories without barium sulphate (left) and with barium sulphate (right).

SSE technology enabled the preparation of small batches of suppositories for daily administration to rats in a single step process and without the need for suppository molds adapted to small animals. The printed suppositories had an acceptable consistency for normal handing and were well-defined. No material slumping was observed during the printing process, and it was not necessary to use a refrigerated build plate to help solidify the printed layers. During the printing process, the syringe temperature was set at 42 °C being carefully controlled to avoid clogging of the nozzle by solidified material. The suppositories were printed horizontally and the printing time for each was 30 s (Figure 2). The mean weight of the 3D printed suppositories was 54.5 ± 4.2 mg. The final drug loading of the suppositories was 0.51 ± 0.04 mg (theoretical amount was 0.50 ± 0.02 mg), suggesting that the drug did not undergo any degradation either during the mixture preparation or printing process.

**Figure 3:**
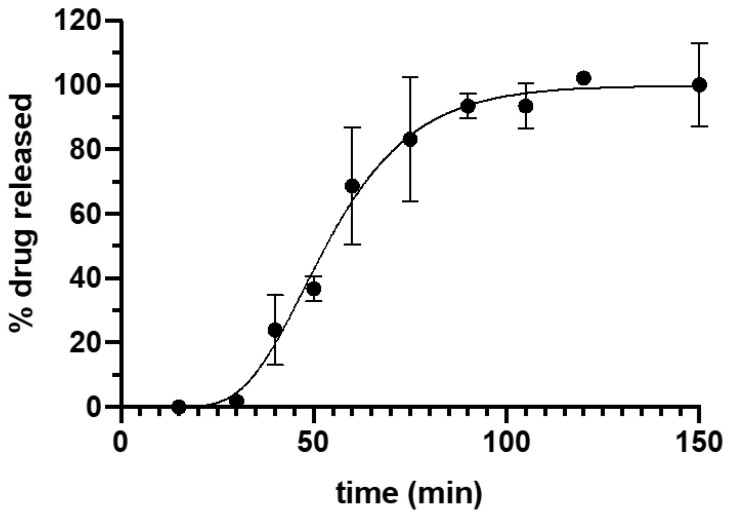
Drug release profile of Gel 44 suppositories in phosphate buffer pH 8. Error bars represent standard deviation (SD).

Tacrolimus dissolution data is shown in Figure 3. The fitting of the experimental data to Equation (1) yielded a high correlation coefficient (R2) = 0.9927. The maximum percentage of drug released was 99.80 ± 2.43 and the minimum 4.41 × 10^−8^ ± 3.29 × 10^−7^, with a k value of 0.065 ± 0.007. The drug release from the suppositories showed a lag time of 15.5 min that could be explained by the time required for the disaggregation of the suppository and the beginning of emulsion formation. Afterwards, there was a continuous release of the drug with more than 50% of tacrolimus released within the first hour, achieving 100% release of the drug at 90 min. The complete release of the drug from the formulation corresponds to the asymptotic part of the curve.

**Figure 4:**
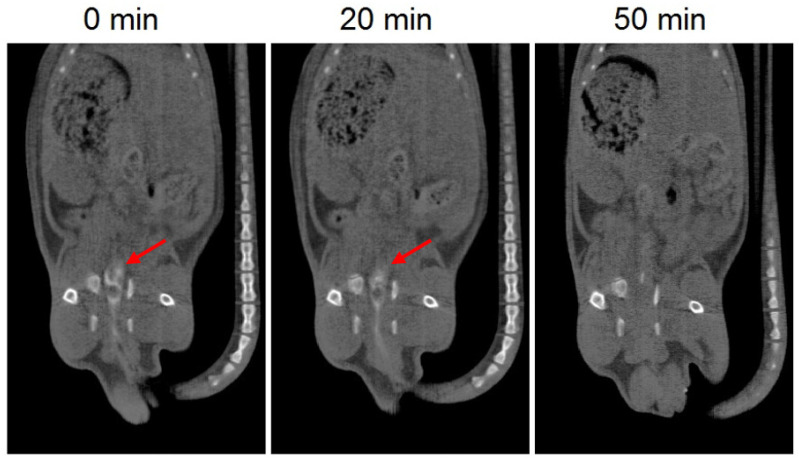
CT images of 3D printed suppositories with barium sulphate at different times. The red arrow indicates the location of the suppository (white color).

The in vivo disintegration time of the 3D printed suppositories was evaluated by CT imaging. Barium sulphate was included in the suppositories as a contrast agent to allow visualization of the devices with CT imaging. Since the composition of the suppositories was affected by the addition of barium sulphate, this test provides an estimated rough measure of the in vivo disintegration time. CT images showed that 20 min post-administration the formulation is still in the colon, which is in agreement with the results of the in vitro release test showing that almost no drug was released after 20 min. On the other hand, the suppository was no longer observed in the CT image of 50 min post-administration, which corresponds to a release of approximately 40% of the drug and indicates that the suppository had already dispersed (Figure 4).

The in vitro disintegration time of the suppositories with and without barium sulphate was evaluated to assess whether the inclusion of barium sulphate in the formulation affects the disintegration time of the suppositories. The mean disintegration time of both suppositories was faster than 10 min (7.1 ± 0.1 min for barium sulphate suppositories and 4.1 ± 1.1 min for suppositories without barium sulphate) and the values were not statistically significantly different (*p* < 0.05). The disintegration times obtained with the in vitro test were markedly different from the disintegration times obtained in vivo. This could be due to the lower amount of liquid medium present in the colon, which delays the complete disintegration of the suppositories.

### 3.2. Assessment of the Efficacy of Tacrolimus Treatment by PET/CT Imaging and Histopathological Analysis

**Figure 5:**
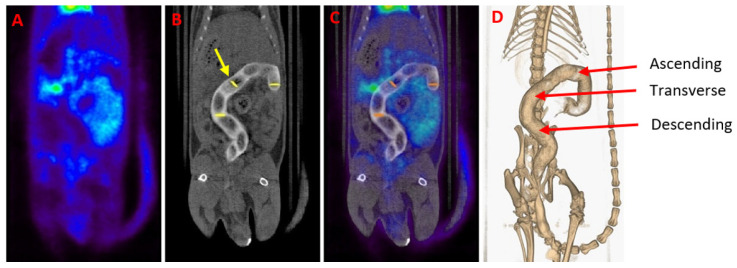
(**A**) Gastrointestinal PET image of a healthy animal. (**B**) CT scan of the same animal. A contrast agent was used for enhancing contrast in the colon of the animal (white color). The yellow arrow indicates one of the circularly delineated regions of interest (ROIs). (**C**) [^18^F]FDG PET/CT image obtained by the fusion of (A) and (B) images. Notice the normal [^18^F]FDG uptake in the heart, bladder and kidneys. (**D**) Three-dimensional colon reconstruction from CT images with the three colonic sections indicated by the red arrow (ascending, transverse and descending).

[^18^F]FDG PET/CT studies were conducted to assess the feasibility of treating experimental IBD with 3D printed suppositories loaded with tacrolimus. PET images were quantified in terms of SUV_max_ values, which are measures of relative [^18^F]FDG tissue uptake, providing data on the evolution of the disease throughout the days. Moreover, CT imaging allows anatomic localization of the colon area through the rectal administration of 1 mL of iopromide (Ultravist 300 mg/mL), a contrast agent that facilitates the delineation of ROIs in fused PET/CT images (Figure 5). The administration of the suppositories and the performance of the [^18^F]FDG PET/CT studies were separated in time to avoid problems with the release of the drug.

**Figure 6:**
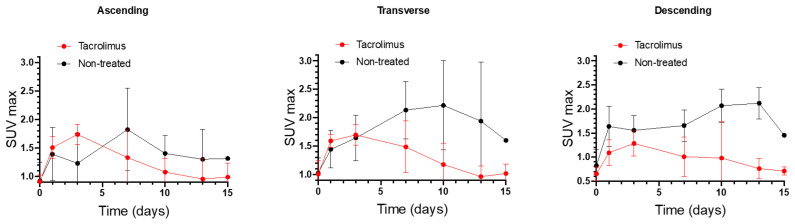
Longitudinal SUV_max_ values for ascending, transverse and descending colonic segments over time for non-treated rats and animals treated with the 3D printed tacrolimus suppositories. Error bars represent standard deviation (SD).

SUV_max_ values of non-treated IBD animals were compared with the SUV_max_ values of IBD animals treated with the 3D printed tacrolimus suppositories. Figure 6 shows separately the SUV_max_ values obtained for ascending, transverse and descending colon sections of treated and non-treated animals. The effect of rectally administered tacrolimus begins to be noticed from the seventh day. The highest remission of inflammation was reached on days 10 and 13 mainly in the transverse and descending colon, with statistically significant differences between the treated and non-treated groups [13].

**Figure 7:**
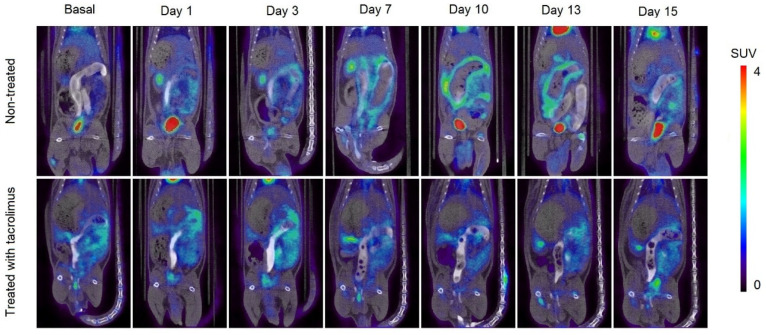
Longitudinal fused PET/CT images over time obtained from rats without treatment (13) after the colitis induction and treated with the 3D printed tacrolimus suppositories. The metabolic activity is coded on a color scale ranging from blue (low [^18^F]FDG uptake) to red (high [^18^F]FDG uptake).

In Figure 7, it can be appreciated the increase in metabolic activity around the colon area due to inflammation caused by IBD, which corresponds to the increase in SUV_max_ values. In the treated group, there is a high [^18^F]FDG uptake around the colon on days 1–3, which is subsequently reduced when the treatment administration begins on day 3. On days 7–13, only a slight [^18^F]FDG accumulation can be appreciated in treated rats. On the contrary, the remission of inflammation in non-treated rats begins from day 15, associated with the spontaneous remission of the TNBS-induced colitis.

**Figure 8:**
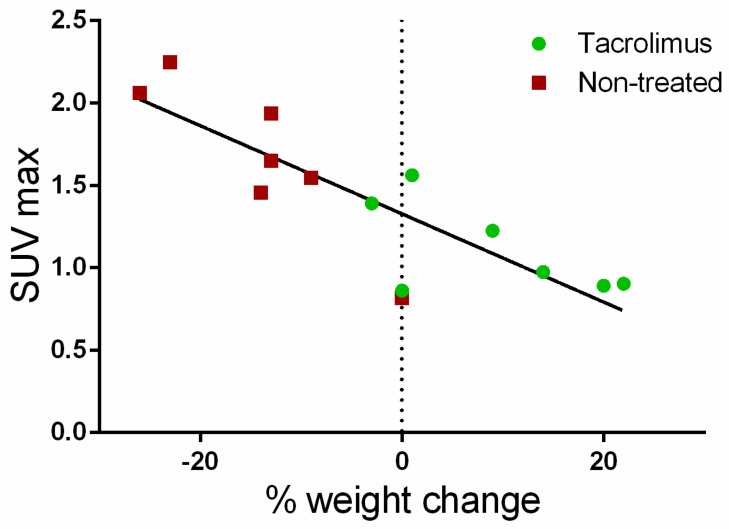
Correlation of average SUV_max_ values with body weight changes from the baseline (dotted line).

Furthermore, a correlation (Pearson coefficient R^2^ = 71.48%) was found between the average SUV_max_ values for the ascending, transverse and descending regions of the colon with weight changes for each day on which PET/CT studies were performed. As can be seen in Figure 8, weight changes with respect to baseline are clearly associated with SUV_max_ values. Positive weight changes are linked to lower SUV_max_ values, which are mainly found in the group treated with tacrolimus suppositories. On the contrary, weight loss is related to the higher SUV_max_ values found in the non-treated group.

**Figure 9:**
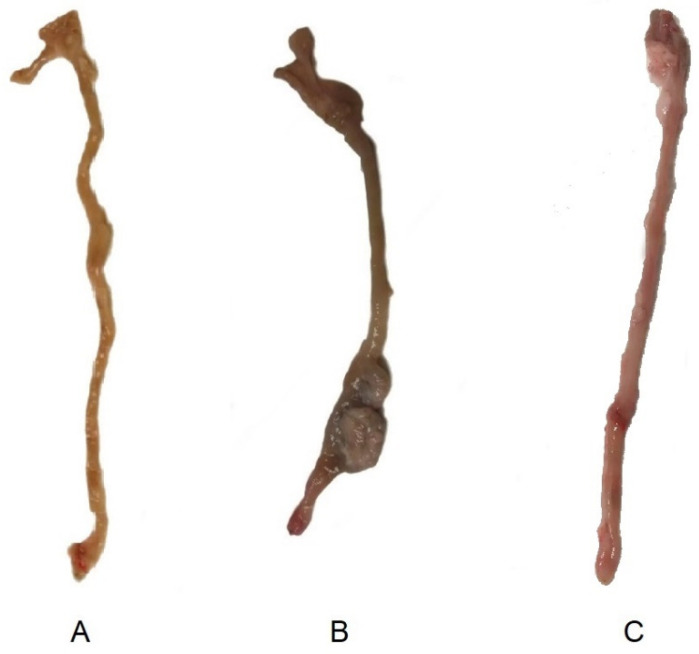
Macroscopic images of ex-vivo colons on day 15 post-TNBS induction in (**A**) control group (healthy animals) (13), (**B**) non-treated inflammatory bowel disease (IBD) rats, and (13) (**C**) IBD rats treated with tacrolimus.

Macroscopic images of ex-vivo colons on day 15 after colitis induction are shown on Figure 9. Images of a non-treated and control animal extracted from our previous study [13] are also shown to be compared with the sample obtained from an animal treated with the tacrolimus 3D printed suppositories. Wall thickening and areas of necrosis are clearly seen in the colon of the non-treated animal, whereas the treated has clearly recovered, with no sign of damage visible to the naked eye.

**Figure 10:**
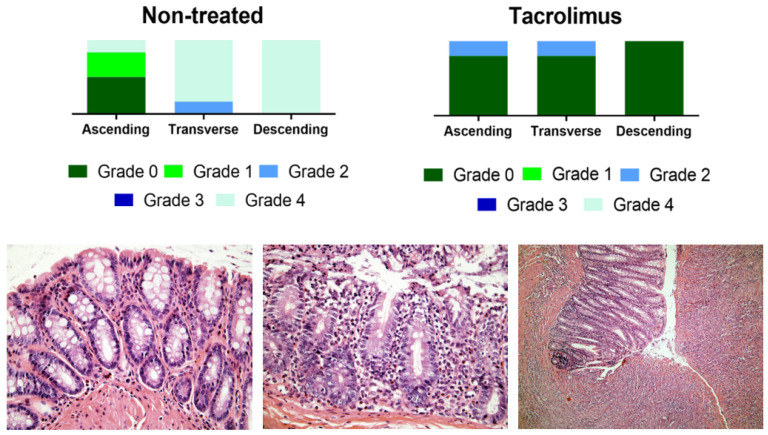
Above, graphical representation of Nancy grades for ascending, transverse and descending colon sections for non-treated and tacrolimus groups. Below, representative optical microscopy images at 40× (left and middle) and 10× magnification (right) of the Nancy index obtained from rats treated with tacrolimus suppositories. On the left, histologically normal mucosa corresponding to grade 0 of the Nancy index. On the middle, note the presence of occasional neutrophils in the lamina propria, with no signs of ulceration, corresponding to a grade 2 of Nancy index. On the right, ulcerated colonic mucosa, with loss of crypts replaced by granulation tissue, corresponding to a grade 4 of the Nancy index [13].

The histological evaluation of colon samples using the Nancy histological index [41] as a reference score showed improved values for the group treated with tacrolimus suppositories compared to the non-treated group. For transverse and descending colon samples, Mann–Whitney test gives a *p* value < 0.05 for differences between treated and non-treated animals [13]. Figure 10 provides the results of the histological evaluation of each colon region on day 15 for non-treated and treated groups. The group treated with tacrolimus suppositories showed grade 0 in 5/5 descending colon, in 4/5 transverse colon and in 4/5 ascending colon samples. Moreover, grade 2 was only found in 1/5 ascending colon and 1/5 transverse colon samples. Figure 10 also shows representative images of different Nancy grades obtained from the histological examination of colon tissue samples from treated and non-treated rats. Different histological changes can be appreciated depending on the degree of colon damage, ranging from normal mucosal structures found in samples rated as grade 0, to relevant mucosal ulceration and granulation tissue found in samples rated as grade 4. In intermediate grades of colon damage, neutrophil infiltration with less or no sign of ulceration can be observed.

For the transverse and descending colon, the Mann–Whitney nonparametric test gives a *p*-value < 0.05 for differences between non-treated and treated animals.

Furthermore, the time it takes for an animal to regain baseline SUV_max_ values (MRRT) and the time at which the maximum value of SUV_max_ occurs (t_max_) were calculated and the results are shown in Table 1 The statistical comparison (two-way ANOVA) indicates that for all colon sections, MRRT and t_max_ values were lower for rats treated with tacrolimus. The average time for a rat to recover baseline SUV_max_ values for the entire colon was 9.22 days for non-treated rats, which was reduced to 5 days after treatment with tacrolimus suppositories. In addition, the mean time required to reach the maximum SUV_max_ value and to begin to decrease was 10.50 days for non-treated animals compared to the 3.1 days for animals treated with tacrolimus. Consequently, the results indicate much faster recovery in animals treated with tacrolimus suppositories.

To compare the efficacy of tacrolimus suppositories with other proposed treatments, MRRT and t_max_ were calculated from previously published data [13,15]. Results are shown in Table 2. No significant differences were obtained between rats treated with tacrolimus suppositories and rats treated with a corticosteroid (0.5 mg/kg methylprednisolone intraperitoneal) or resveratrol (10 mg/kg intrarectal), but lower MRRT and t_max_ values were observed compared to melatonin.

On the whole, the decrease in the disease activity assessed by PET/CT imaging and histological analysis confirmed the therapeutic effect of treatment with tacrolimus suppositories. The treatment with the rectal formulations achieves a disease remission in 5 days, compared to the 9 days for the non-treated group [13]. This therapeutic effect was comparable to that obtained with the administration of an intraperitoneal corticosteroid or resveratrol in the form of an enema. The fact that treatment with tacrolimus has obtained a therapeutic effect similar to that of resveratrol can be explained by the antioxidant effect of the latter [42], since it is known that oxidative stress plays a role in the pathogenesis of IBD [43].

The therapeutic activity of tacrolimus has been previously described in other animal studies, in which tacrolimus was administered orally in the form of microparticles [44] and microspheres [45] prepared with pH sensitive polymers to allow drug delivery to the colon and nanoparticles [17] with enhanced adhesion to the inflamed tissue. Thus, the present study was focused on the development of a rectal dosage form that delivers the incorporate drug content directly to the colon, avoiding the side effects derived from an undesired early drug release in upper parts of the GI tract. The capability of SSE 3DP to fabricate lipid-based printlets [31] was exploited to produce small suppository-shaped formulations loaded with the exact amount of drug needed for the animals. Therefore, this method has been shown its potential to prepare formulations considering each patient and required doses. The selected mixture of lipid excipients, being Gelucire 44/14 the main component, has demonstrated adequate printability properties. The disintegration time and the time required for emulsion formation from rectal devices prepared with this mixture were also acceptable. The local drug release was confirmed by CT images, which allowed the location of the suppositories after rectal administration and gave an approximate measure of the in vivo disintegration time. The self-emulsifying suppositories disintegrated in an acceptable amount of time, even with the lack of fluid in the colon. Since this drug delivery approach allows the release of the drug in the inflamed tissue area, a reduction of adverse effects of tacrolimus could be obtained due to a more local effect of the drug.

The manufacturing opportunities that 3DP could offer in preclinical research have not yet been fully exploited [46,47,48]. This highly flexible technology allows the on-demand manufacturing of devices containing the exact dosage of the drug and with sizes and geometries adapted to the animal model. To this date, few studies have reported the use of 3DP in preclinical research, with almost all focusing on oral drug delivery [49,50,51,52,53]. The study herein is the first that uses 3DP to manufacture dosage forms for rectal administration in rats. The results yielded by this work confirm the feasibility of this technique to prepare drug loaded lipid-based suppositories with self-emulsifying properties and adequate characteristics for its administration to rats. Moreover, the combination of lipid excipients used in this work has proved to be a suitable carrier for tacrolimus, which was successfully solubilized and released from the lipid droplets of the formed emulsion.

## 4. Conclusions

The versatility of semisolid extrusion 3DP allowed the preparation of small self-emulsifying suppositories containing an exact dose of the drug with a size and shape adapted for their rectal administration in rats. The combination of Gelucire 44/14 and coconut oil showed adequate printability, and the disaggregation of the suppositories and subsequent release of the drug from the formed emulsion occurred within an acceptable period of time (more than 50% of tacrolimus released within the first hour). Moreover, the remission of the disease was achieved in five days, compared to the 9 days required for the non-treated animals to recover. Moreover, [^18^F]FDG PET/CT imaging was shown to be a useful tool for the assessment of the disease over time and CT imaging allowed for a better in vivo characterization of the suppositories. The quantification of inflammation using the SUV_max_ parameter, commonly used in clinical practice, helps the translation into humans of this new therapeutic approach. The combination of semisolid extrusion 3DP with medical imaging offered a new approach for the development and assessment of novel IBD therapies in the preclinical area.

## Figures and Tables

**Table 1:** Comparison of the mean rat recovering time (MRRT) and t_max_ values of SUV_max_ vs. time with and without tacrolimus treatment.

	Non-Treated				Tacrolimus			
	MRRT (days)		t_max_ (days)		MRRT (days)		t_max_ (days)	
	Mean	SD	Mean	SD	Mean	SD	Mean	SD
Ascending	9.76	2.62	10.00	3.00	4.78	0.81	4.00	2.00
Transverse	8.37	1.01	10.75	2.87	4.68	1.37	3.50	2.52
Descending	9.54	1.06	10.75	1.50	5.74	1.86	1.80	1.10
Mean ± SD	9.22	0.61	10.50	0.35	5.07	0.48	3.10	0.94

**Table 2:** MRRT and t_max_ values of rats treated with the corticosteroid methylprednisolone, resveratrol and melatonin. The parameters were calculated from data from previously published works.

		Methylprednisolone		Resveratrol		Melatonin	
		Mean	SD	Mean	SD	Mean	SD
MRRT (days)	Ascending	4.57	1.5	4.17	1.97	6.36	1.53
	Transverse	5.37	1.12	4.49	1.79	7.42	2.27
	Descending	4.29	1.53	3.72	2.49	6.37	1.5
	Mean ± SD	4.74	0.56	4.13	0.39	6.72	0.61
t_max_ (days)	Ascending	3.00	2.83	1.83	1.03	5.92	4.10
	Transverse	3.25	1.26	1.92	1.00	4.75	3.49
	Descending	3.00	0.01	1.50	0.90	4.50	3.06
	Mean ± SD	3.08	0.14	1.75	0.22	5.06	0.76
Data obtained from		[13]		[15]			
